# Amaryllidaceae Alkaloid Cherylline Inhibits the Replication of Dengue and Zika Viruses

**DOI:** 10.1128/AAC.00398-21

**Published:** 2021-08-17

**Authors:** Seydou Ka, Natacha Merindol, Aïssatou Aïcha Sow, Amita Singh, Karima Landelouci, Mélodie B. Plourde, Geneviève Pépin, Marco Masi, Roberta Di Lecce, Antonio Evidente, Matar Seck, Lionel Berthoux, Laurent Chatel-Chaix, Isabel Desgagné-Penix

**Affiliations:** a Department of Chemistry, Biochemistry, and Physics, Université du Québec à Trois-Rivières, Trois-Rivières, Québec, Canada; b Centre Armand-Frappier Santé Biotechnologie, Institut National de la Recherche Scientifique, Laval, Québec, Canada; c Department of Medical Biology, Université du Québec à Trois-Rivières, Trois-Rivières, Québec, Canada; d Dipartimento di Scienze Chimiche, Universita’ di Napoli Federico II, Complesso Universitario Monte Sant’Angelo, Naples, Italy; e Laboratoire de Chimie Organique et Chimie Thérapeutique, Faculté de Médecine, de Pharmacie, et d’Odontologie de Dakar, Dakar, Senegal

**Keywords:** Amaryllidaceae, alkaloids, flavivirus, cherylline, antivirals, dengue virus, Zika virus, lycorine

## Abstract

Dengue fever, caused by dengue virus (DENV), is the most prevalent arthropod-borne viral disease and is endemic in many tropical and subtropical parts of the world, with an increasing incidence in temperate regions. The closely related flavivirus Zika virus (ZIKV) can be transmitted vertically *in utero* and causes congenital Zika syndrome and other birth defects. In adults, ZIKV is associated with Guillain-Barré syndrome. There are no approved antiviral therapies against either virus. Effective antiviral compounds are urgently needed. Amaryllidaceae alkaloids (AAs) are a specific class of nitrogen-containing compounds produced by plants of the Amaryllidaceae family with numerous biological activities. Recently, the AA lycorine was shown to present strong antiflaviviral properties. Previously, we demonstrated that Crinum jagus contained lycorine and several alkaloids of the cherylline, crinine, and galanthamine types with unknown antiviral potential. In this study, we explored their biological activities. We show that *C. jagus* crude alkaloid extract inhibited DENV infection. Among the purified AAs, cherylline efficiently inhibited both DENV (50% effective concentration [EC_50_], 8.8 μM) and ZIKV replication (EC_50_, 20.3 μM) but had no effect on HIV-1 infection. Time-of-drug-addition and -removal experiments identified a postentry step as the one targeted by cherylline. Consistently, using subgenomic replicons and replication-defective genomes, we demonstrate that cherylline specifically hinders the viral RNA synthesis step but not viral translation. In conclusion, AAs are an underestimated source of antiflavivirus compounds, including the effective inhibitor cherylline, which could be optimized for new therapeutic approaches.

## INTRODUCTION

Dengue fever is a viral disease caused by dengue virus (DENV), a flavivirus related to Zika virus (ZIKV) and belonging to the family Flaviviridae. Both viruses are transmitted to humans through biting of female mosquitoes, Aedes aegypti or Aedes albopictus (1). DENV and ZIKV possess an ∼11-kb-long positive-sense stranded RNA genome encoding capsid (C), membrane precursor (prM), and envelope (E) structural proteins and NS1, NS2A, NS2B, NS3, NS4A, NS4B, and NS5 nonstructural proteins (2–4). To date, four serotypes of DENV (DENV1 to -4) are described as the cause of dengue hemorrhagic fever and dengue shock syndrome, both being potentially fatal (5, 6). Nearly four billion individuals are at risk of contracting dengue fever. An estimated 400 million people catch it every year. Between 50 and 100 million human symptomatic cases are reported annually in tropical and subtropical regions, with a yearly death toll of 20,000 individuals, most of them being children (7), posing a considerable threat for public health in over 100 countries (8). The number of cases is constantly increasing. From 2017 to 2018, two outbreaks associated with DENV-1 occurred in Senegal (9), and DENV is now present in more temperate regions such as southern Europe.

ZIKV infection is mostly asymptomatic or associated with mild symptoms in adults, but it can cause Guillain-Barré syndrome (10, 11). Fetal ZIKV infection causes more serious conditions, such as the congenital Zika syndrome and other severe birth defects. During the 2015 ZIKV outbreak in Brazil, 4,300 children were born with microcephaly (10).

To date, there are no approved antiviral therapies against DENV or ZIKV and no vaccine for ZIKV (12). In the case of DENV, the efficacy of the approved tetravalent vaccine Dengvaxia is not optimal for all serotypes, and its use is not recommended for DENV-seronegative individuals (13, 14). Therefore, effective compounds inhibiting ZIKV and DENV replication are urgently required, and drugs with broad-spectrum activity against several pathogenic flaviviruses would definitely constitute an asset in the therapeutic arsenal.

Medicinal plants such as Amaryllidaceae contain alkaloids with various biological activities, including antimosquito activity against Aedes aegypti and antiviral properties (15–19). The Amaryllidaceae Crinum macawonii methanolic extract inhibits *in vitro* infection of yellow fever virus (YFV) and Japanese encephalitis virus (JEV), two flaviviruses (20). Aqueous and organic extracts from Crinum jagus contain molecules exhibiting anti-inflammatory (21), antibacterial (22), and antienteroviral activities (23), while their effect on flavivirus replication remains unknown. We isolated the Amaryllidaceae alkaloids (AAs) lycorine, sanguinine, crinine, gigancrinine, flexinine, cherylline, gigantelline, and gigantellinine and hippadine (an amide close to lycorine) from *C. jagus* (*giganteum*) and showed that some display cytotoxic activity and antiacetylcholinesterase potential (24). In preceding studies, lycorine was shown to inhibit *Flaviviridae* such as the flaviviruses DENV, ZIKV, YFV, and JEV and several viruses from other families, including *Retroviridae* (HIV-1) and *Coronaviridae*, as well as DNA viruses (25–32). Except for lycorine, the antiviral potential of AAs has been poorly studied. Continuing our screening of biological activities of native and understudied Amaryllidaceae from Senegal, we investigated *C. jagus’*s alkaloid potential. We hypothesized that *C. jagus* extract displays antiflaviviral properties and that it contains alkaloids with antiflaviviral potential in addition to lycorine.

In this study, we assessed the *in cellulo* antiviral activity of AAs isolated from *C. jagus* and identified cherylline as a novel inhibitor of the DENV and ZIKV life cycles. Using time-of-drug-addition and -removal assays, as well as modified DENV genomes, we further showed that the viral RNA replication step of the DENV life cycle is the main target of cherylline. This comprehensive assessment uncovers cherylline as a new natural product candidate to be optimized for the development of therapeutics to fight flavivirus infections.

## RESULTS

### *Crinum jagus* alkaloid extract displays anti-DENV activity.

**FIG 1:**
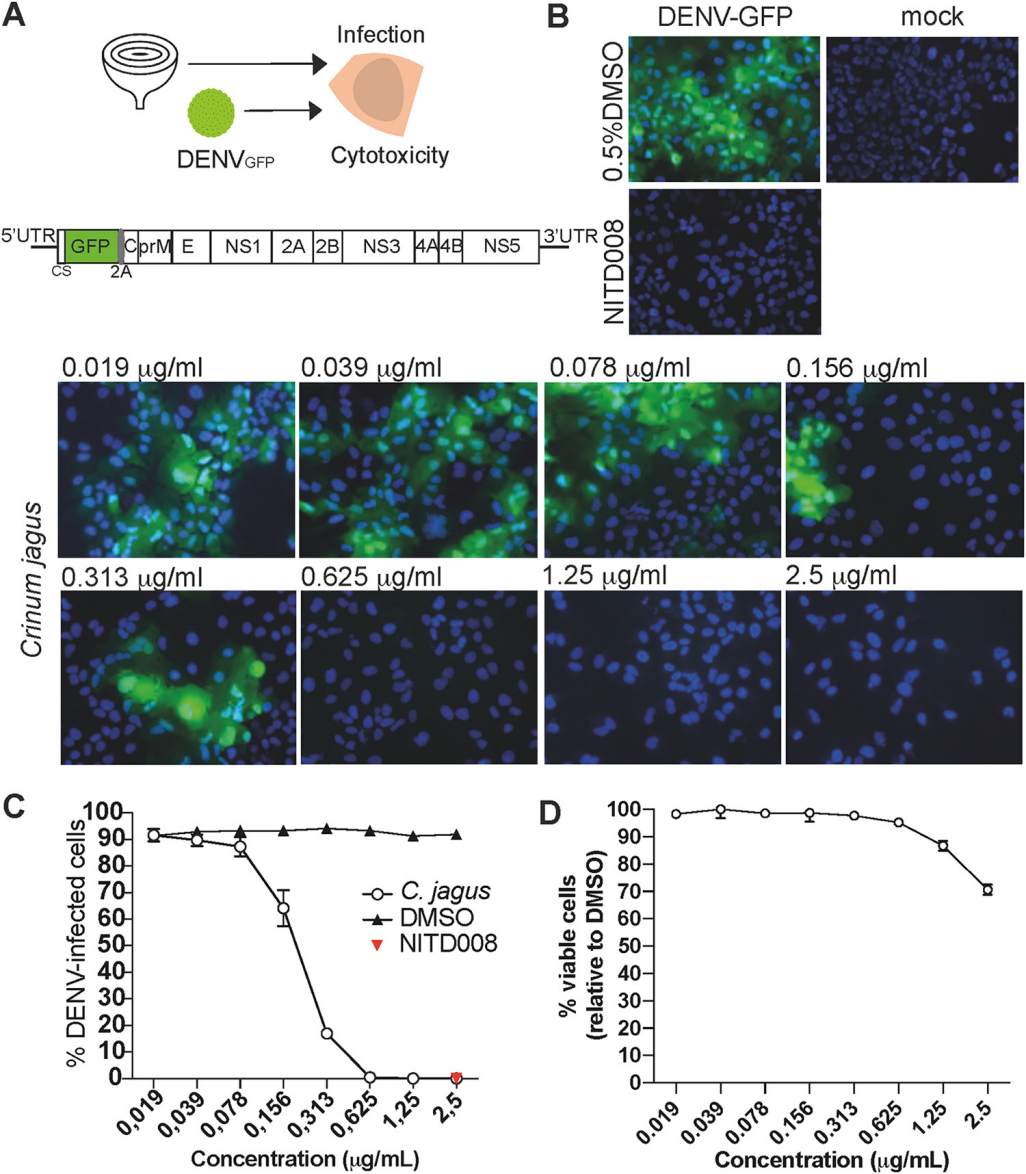
*Crinum jagus* alkaloid extract’s anti-DENV activity. (A) Schematic experimental design of the antiviral assay using *Crinum jagus* bulbs and DENV, with DENV_GFP_ construct genome representation. In this system produced by Fischl and Bartenschlager (33), 103 nucleotides of DENV capsid gene were duplicated and cloned upstream of *gfp*, which was then fused to the capsid gene with a 2A peptide (82). *Gfp* is replicated as part of the viral genome and translated as a component of the polyprotein. The 2A peptide allows GFP to be released from DENV polyprotein during or following the translation process. (B) Inhibition of DENV_GFP_ infection with *C. jagus* alkaloid extract observed by inverted microscopy in Huh7 cells. Representative images are shown with cell nuclei stained with Hoechst33342 (blue) and DENV infection (green). (C) Anti-DENV activity of *C. jagus* crude extracts. The inhibition of DENV_GFP_ infection in Huh7 cells by *C. jagus* alkaloid extract dilutions was measured by flow cytometry. (D) Cytotoxicity of *C. jagus* bulbs crude alkaloid extract in Huh7 cells as measured by the XTT [2,3-bis-(2-methoxy-4-nitro-5-sulfophenyl)-2H-tetrazolium-5-carboxanilide salt] assay.

First, we evaluated the effect of *C. jagus* extract on the replication of DENV. We used green fluorescent protein (GFP)-expressing reporter DENV particles for infection (Fig. 1A to C). In this system, the GFP coding sequence is included in the unique open reading frame of the viral RNA genome; 103 nucleotides of DENV capsid gene are duplicated and cloned upstream of *gfp*, fused to the capsid gene with a 2A peptide. *Gfp* is translated in-frame with the polyprotein. The 2A peptide allows GFP to be proteolytically released from DENV polyprotein during or shortly after translation (33). Hence, GFP fluorescence is directly proportional to the extent of polyprotein production. This allows the detection of viral replication in infected cells using microscopy or flow cytometry. The inhibitory properties of *C. jagus* crude extract were evaluated in Huh7 hepatocarcinoma cells, a classical model for flavivirus study. NS5 RNA polymerase inhibitor NITD008 was used as a positive control (34, 35). At concentrations ranging from 0.078 to 2.5 μg/ml, DENV_GFP_ replication was significantly inhibited by the extract (Fig. 1B and C). Viral inhibition followed a dose-dependent response with an 50% effective concentration [EC_50_] of 0.25 μg/ml (Fig. 1C). At 0.625 μg/ml, no infection could be detected by either flow cytometry or microscopy. We also verified the cytotoxicity of *C. jagus* crude extract antiviral concentrations in Huh7 cells. Extracts were weakly cytotoxic at all concentrations, with a minimum of 71% of viable cells at the highest concentration of 2.5 μg/ml. At 0.625 μg/ml, 100% of the cells were viable, while 0% were infected (Fig. 1D).

**FIG 2:**
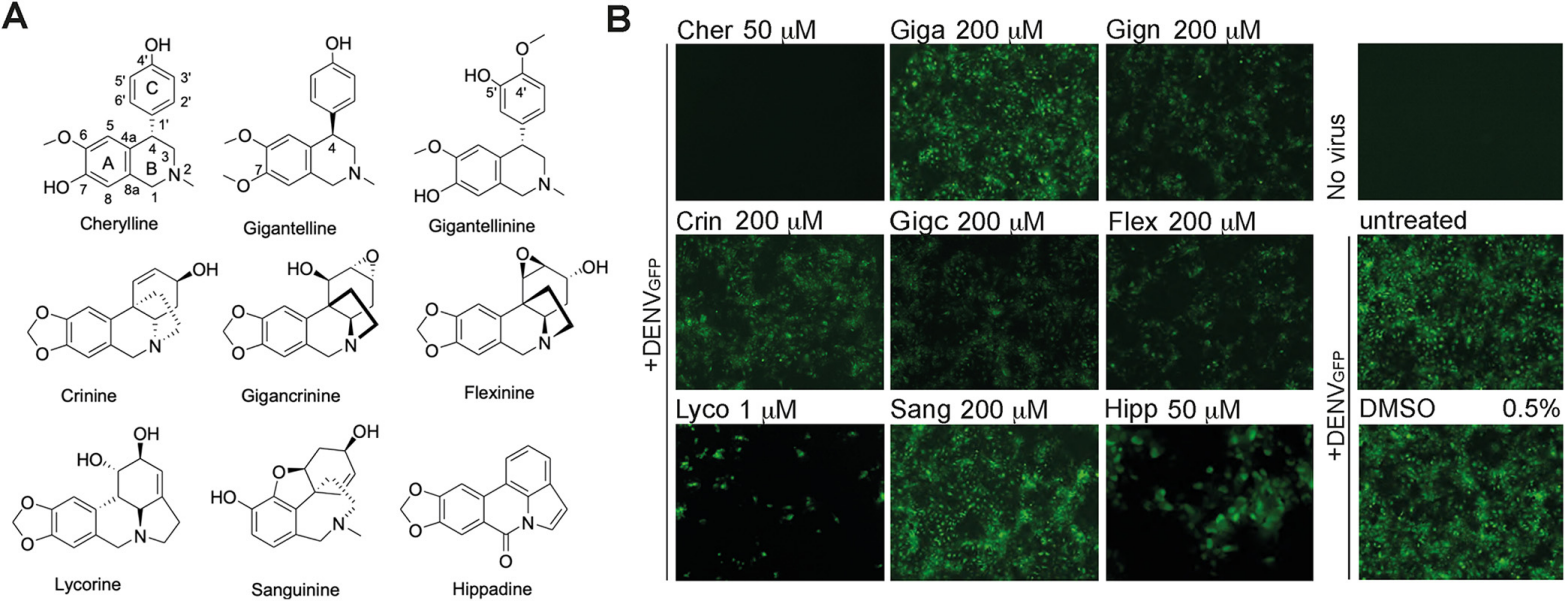
Screening of the anti-DENV activity of *C. jagus* isolated Amaryllidaceae alkaloids (AAs). (A) Structures of the nine AAs isolated from bulbs of *C. jagus*. Cherylline-type alkaloids comprised cherylline, gigantelline, and gigantellinine, while crinine types comprised crinine, gigancrinine, and flexinine. Lycorine, sanguinine, and hippadine were also isolated. (B) Anti-DENV activity of AAs. Huh7 cells were treated with compounds (AAs or DMSO vehicle) for 2 h and then infected with DENV_GFP_ at an MOI of 0.15 for 72 h. Infectivity was visualized as GFP^+^ cells on an inverted microscope system with ×5 and ×20 (hippadine) objectives. The experiment was performed in triplicates at two dilutions. At least 3 pictures were taken from each well. One representative picture of the lowest dilution is displayed. Cher, cherylline; Giga, gigantelline; Gign, gigantellinine; Crin, crinine; Gigc, gigancrinine; Flex, flexinine; Lyco, lycorine; Sang, sanguinine; Hipp, hippadine.

We next wanted to gain further knowledge into which AA present in *C. jagus* extract was responsible for the anti-DENV activity. Thus, we studied the nine alkaloids isolated from *C. jagus*, i.e., three cherylline-type (cherylline, gigantelline, and gigantellinine), three crinine-type (crinine, gigancrinine, and flexinine) one galanthamine-type (sanguinine), lycorine, as the sole representative of its own type, and hippadine (Fig. 2A). Excluding lycorine, the antiflaviviral abilities of these families of AAs are unknown. Huh7 cells were treated with AAs and infected with DENV_GFP_ at a multiplicity of infection (MOI) of 0.15, 2 h later. Infection was visualized at 72 h postinfection (hpi) by detecting GFP using microscopy (Fig. 2B). Treatment with lycorine caused a sharp decrease in DENV-GFP-infected cells, confirming its strong anti-DENV activity. Interestingly, several other AAs inhibited DENV_GFP_ infection. A notably strong antiviral effect was observed in wells in which cells were treated with 50 μM cherylline, with no detectable infection (Fig. 2B; see Fig. S1 in the supplemental material). Other AAs displayed antiviral activity with an efficiency ranging from moderate (hippadine, flexinine, and gigantellinine) to weak (gigantelline) to very weak (sanguinine) at the chosen concentration. Interestingly, AAs of the same ring type with very similar structure, such as cherylline, gigantelline, and gigantellinine, presented very distinct strengths of inhibition on DENV_GFP_ infectivity. Thus, differences in biological activity were not associated with a specific ring type.

### Cherylline is a potent inhibitor of DENV.

Based on the antiviral activity screening from Fig. 2, we pursued the characterization of the four alkaloids with the highest anti-DENV activity.

**FIG 3:**
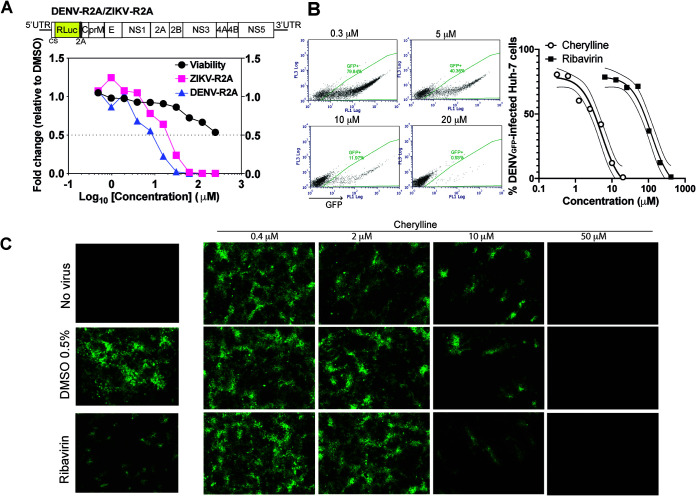
Cherylline displays anti-DENV activity. (A) Impact of cherylline treatment on DENV_R2A_ (purple) and ZIKV_R2A_ (pink) replication was tested in Huh7.5 cells (MOI, 0.001), and viral-dependent luciferase luminescence was measured at 48 hpi. Cell viability (ATP) was assessed at 48 h. Results are displayed as fold changes in viability and replication, with 1 meaning no change compared to matched concentrations of DMSO-treated cells. (B) Treatment of Huh7 with cherylline dampened infectivity of DENV_GFP_ replication (MOI, 0.15) in a dose-dependent manner, as measured by flow cytometry 72 hpi. (Left) representative dot plots; (right) percentage of infected Huh7 cells using 0.6 to 100 μM cherylline and 10 to 500 μM ribavirin as a positive control. The nonlinear regression curve fit is shown with the 95% of confidence interval in finer lines. (C) Representative pictures of Huh7 treated with different concentrations of cherylline and infected with DENV_GFP_ at an MOI of 0.15. Ribavirin was used as a positive control, and DMSO as a negative control. Infected cells were visualized on an inverted microscope system at 72 hpi.

The antiviral activity of cherylline, hippadine, gigantellinine, and flexinine was studied using DENV_R2A_ construct infection in Huh7.5 cells (Fig. 3A; Fig. S2A). DENV_R2A_ is similar to DENV_GFP_, but *Renilla luciferase* replaces *gfp* as the reporter gene. Infection levels were measured at 48 hpi. Gigantellinine and flexinine anti-DENV activity required high concentrations (EC_50_, 104.6 and 63.4 μM, respectively; Fig. S2A). Hippadine antiviral activity on DENV_R2A_ was difficult to distinguish from its cytopathic effect (EC_50_, 73.8; 50% cytotoxic concentration [CC_50_], 175.9 μM; stimulation index [SI], 2.38) (Fig. S3; Table 1). Cherylline inhibited DENV_R2A_ replication at an EC_50_ of 8.8 μM (Fig. 3A). The cherylline CC_50_ was not reached at the highest tested concentration (250 μM). Its therapeutic selectivity index cannot be calculated but is predicted to be >28 (Table 1). Furthermore, the inhibitory activity of cherylline, our best candidate, was assessed by infecting Huh7 cells with DENV_GFP_ in the presence of increasing concentrations (from 0.6 to 100 μM) of compound (Fig. 3B and C). Interestingly, cherylline was more potent than the guanosine analogue ribavirin at counteracting DENV_GFP_ infection, with an EC_50_ of8 μM, similar to its effect on DENV_R2A_, compared to an EC_50_of 100 μM for ribavirin. By microscopy, no infected cells were observed at 50 μM, confirming our first experiment. Several foci of infected cells became visible at 10 μM, whereas most of the cells were infected at 2 μM, and there was no apparent difference with the control at 0.4 μM.

**TABLE 1:** EC_50_, CC_50_, and selectivity index of AAs as calculated with GraphPad Prism^*a*^

Alkaloids	CC_50_ (μM)	EC_50_ (μM) for		SI	
		DENV_R2A_	ZIKV_R2A_	DENV_R2A_	ZIKV_R2A_
Lycorine	14.5	0.16	0.41	90.6	35.4
Cherylline	>250	8.8	20.33	>28	>12.3
Hippadine	175.9	73.8	114	2.38	1.54
Flexinine	>250	63.4	216.3	>3.9	>1.15
Gigantellinine	>250	104.6	>250	>2.3	ND

a EC_50_ was determined using DENV/ZIKV_R2A_ infection from 0.05 to 25 μM for lycorine and NITD008 and from 0.5 to 250 μM for the others as in Fig. 2A. CC_50_ was calculated using the same concentrations in the same cell type, Huh7.5 at 48 h posttreatment. CC, cytotoxic concentration; EC, effective concentration; SI, therapeutic selectivity index; ND, not detected.

**FIG 4:**
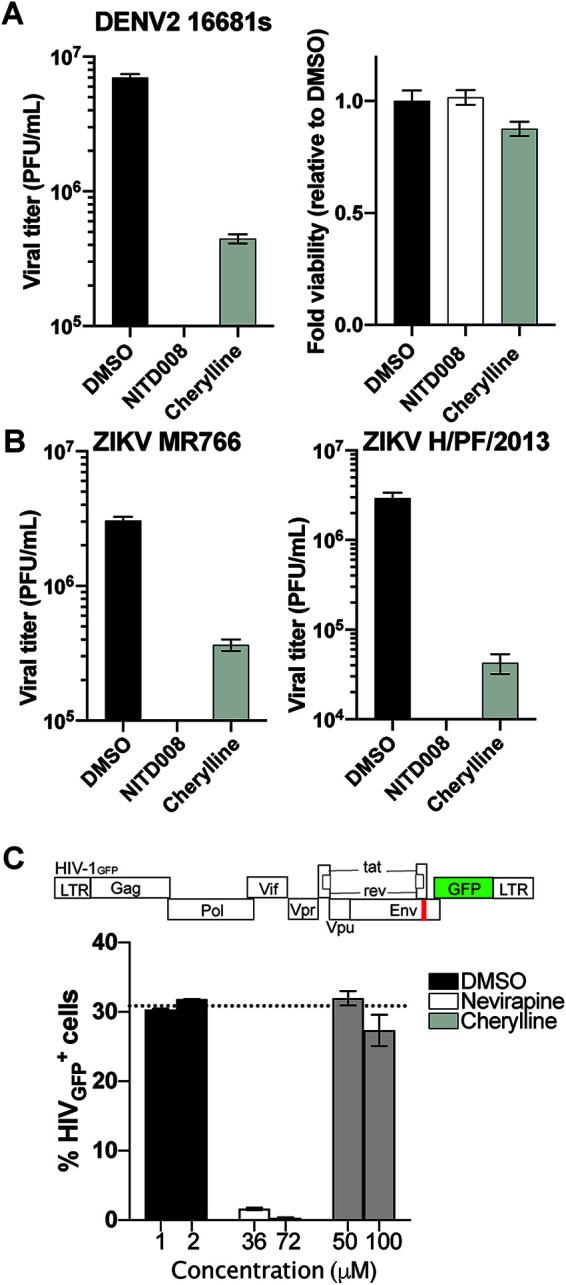
Cherylline displays antiviral activity against DENV and ZIKV. (A) Viral titers were measured by plaque assay on Vero E6 cells using the cytopathic WT DENV2 16881s strain. Huh7.5 cells were infected and treated 2 hpi with compounds. Then, 48 h later, supernatants were harvested and plated on Vero E6 cells (left). Fold changes in viability (ATP) were calculated at 48 hpi in Huh7.5 cells (right). (B) Viral titers were measured as in panel A on Vero E6 cells using cytopathic WT Uganda MR766 ZIKV strains (left) and French Polynesia H/PF/2013 (right). (C) THP-1 cells were treated with two concentrations of each compound in triplicates for 2 h and infected with VSV-G pseudotyped HIV-1GFP at an MOI of 1. The antiretroviral nevirapine was used as a positive control, and DMSO (vehicle), as negative a control. Results were analyzed by flow cytometry 72 hpi.

To validate the antiviral properties of cherylline, we tested its ability to prevent the production of infectious particles using wild-type viruses instead of reporter infectious systems. We infected Huh7.5 cells with viral strain DENV2 16881s, and then treated them with 50 μM of AAs 2 hpi (Fig. 4A). Then, 48 h later, cell supernatants were harvested, and extracellular infectious titers were determined using plaque assays. In parallel, viability was monitored. As expected, NITD008 completely hindered the production of infectious particles (Fig. 4A; Fig. S2B). Cherylline induced a 17-fold decrease in DENV 16881s viral titers (Fig. 4A; Fig. S2B). In contrast, hippadine, gigantellinine, and flexinine treatments exhibited no effects on DENV production (Fig. S2B). In conclusion, cherylline holds a notable anti-DENV potential.

### Cherylline efficiently inhibits ZIKV.

Next, we explored if the isolated AAs were also active on a closely related flavivirus. We examined the impact of cherylline, hippadine, gigantellinine, and flexinine treatment on ZIKV_R2A_ replication in Huh7.5 cells (Fig. 3A; Fig. S2A). Cherylline interfered with the replication of ZIKV at noncytotoxic concentrations, with an EC_50_ of 20.3 μM and a selectivity index of >12.3 (Fig. 3A, Table 1). Hippadine, gigantellinine, and flexinine did not significantly impact ZIKV_R2A_ replication (Fig. S2A).

To validate the anti-ZIKV properties of cherylline, we tested its ability to prevent the production of infectious particles using wild-type viral strains ZIKV H/PF/2013 and ZIKV MR766 instead of reporter infectious systems, as previously described. NITD008 completely hindered the production of infectious particles for both ZIKV strains (Fig. 4B; Fig. S2B). Cherylline diminished the pathogenic ZIKV H/PF/2013 strain viral titer by 100-fold and that of ZIKV MR766 by 88% (Fig. 4B; Fig. S2B).

In contrast, hippadine treatment resulted in a moderate 3-fold (70%) decrease in ZIKV H/PF/2013 viral titers, while gigantellinine and flexinine treatments exhibited marginal effects on ZIKV production (Fig. S2B). In conclusion, the plaque assay using wild-type (WT) viruses indicated that cherylline impedes the DENV2 and ZIKV life cycles.

### Cherylline does not inhibit HIV-1 infection or the interferon response.

Lycorine has been described as a broad antiviral agent, inhibiting many viruses, including flaviviruses and retroviruses. To get insight into the specificity of cherylline antiviral spectrum, we examined its effect on VSV-G-pseudotyped-HIV-1_GFP_ vector (Fig. 4C). This vector does not enter through fusion into cytoplasm, but rather, through endocytosis. It undergoes an incomplete single-cycle infection that includes uncoating, reverse transcriptase, nuclear transport, provirus integration, virus gene transcription, and mRNA translation. With the exception of Env and Nef, viral proteins are produced but do not assemble together to form new virions. Cherylline did not affect HIV-1 infection, nor did any of the AA tested (Fig. S2C). We also determined that cherylline did not trigger a significant amount of type I interferon (IFN) through monitoring of interferon-sensitive response element (ISRE) transcription compared to control treatments (Fig. S3A). These results demonstrate that cherylline specifically targets several flaviviruses with less cytotoxicity than lycorine (Table 1).

### Cherylline targets DENV RNA replication.

We next investigated which step of the DENV life cycle was inhibited by cherylline. DENV viral replication kinetics have been widely studied (36). First, we performed a time course measurement of infection at 12, 24, 48, and 72 h postinfection with DENV_GFP_ (MOI, 0.5) and DENV_R2A_ (MOI, 0.005) (Fig. S4A and B). Cherylline inhibition of viral infection was measured through inhibition of GFP-infected cells (percentage), decrease of GFP and luciferase expression levels (mean fluorescence intensity and luminescence, respectively). A cherylline-induced decrease in viral RNA levels at 12 and 24 h during the first cycle of replication was confirmed using DENV_GFP_ (MOI, 0.5) and WT viruses DENV2 16881s and ZIKV H/PF/2013 at an MOI of 2 (Fig. S4C), although the decrease in viral RNA (vRNA) was more pronounced in the case of DENV_GFP_ and DENV2 16881s. As expected, vRNA levels decrease at later times, which is consistent with Renilla luciferase (Rluc) and GFP assays. The diminution of vRNA at 24 h (i.e., at early time points at which there is no contribution of viral spread in the assay) suggests an effect on the steps prior to the production of viral particles.

**FIG 5:**
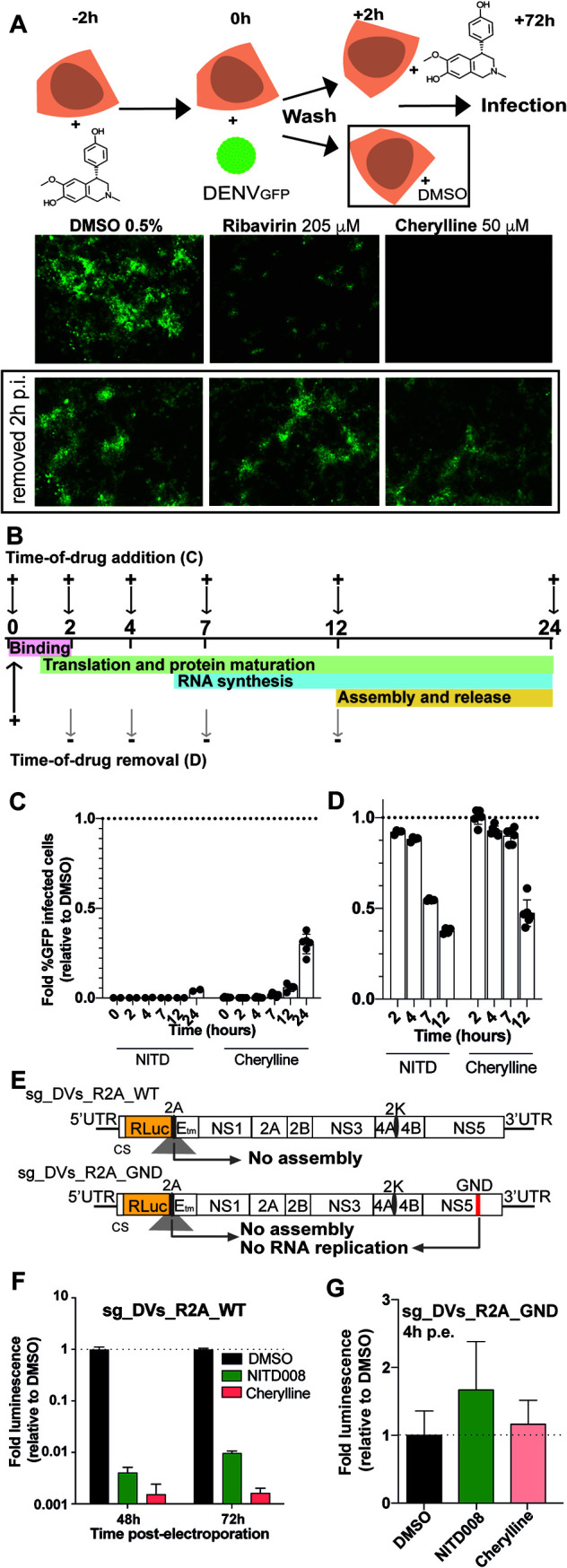
Cherylline blocks DENV replication during RNA synthesis. (A) Infectivity of DENV in Huh7 cells continuously treated with cherylline compared to cells treated only for 2 hpi. DMSO was used as a negative control, and ribavirin as a positive antiviral control. Representative pictures taken at ×5 with an inverted microscope system are shown. (B) Schematic explanation of time-of-drug-addition and -removal rationale. +, addition of compound; –, removal. (C) Time of drug addition. (D) Time of drug removal. For panels C and D, DENV_GFP_ was used at an MOI of 0.1; DMSO was used as a negative control, and the fold infectivity relative to the control was calculated; 1-fold means that the level of infection is the same as control. NITD008 (NITD) was used at 10 μΜ as a positive antiviral control targeting RNA synthesis. Cherylline was used at 50 μΜ. The impact of addition and removal of NITD and cherylline at 0, 2, 4, 7, 12, and 24 hpi on the percentage of DENV_GFP_-infected Huh7 cells was monitored by flow cytometry at 72 hpi. Experiments was performed in triplicates twice. Means with the standard error of the mean (SEM) are shown. (E) Schematic representation of subgenomic replicon sg-DVs_R2A_WT and subgenome sg-DVs_R2A_GND used in panels F and G. Sgs were transfected into Huh7.5 cells, which were then treated with DMSO, NITD008 (5 μΜ), or cherylline (50 μΜ) at the indicated time. (F) Luciferase levels were quantified at 48 and 72 h postelectroporation (hpe) with sg_DVs_R2A_WT and normalized over levels detected in DMSO-treated cells. (G) Luciferase levels were quantified at 4 hpe with RdRp-deficient sg_DVs_R2A_GND and normalized over levels in cells treated with DMSO.

Timing of infection can be precisely associated with viral steps (Fig. 5A and B). Binding and virus entry occur during the first 2 h of viral addition (37). First, cells were treated with alkaloids 2 h prior to infection. We compared infection levels in cells with continuous treatment versus cells in which cherylline was removed 2 hpi (Fig. 5A). DMSO was used as a negative control. The guanosine analogue ribavirin was added as a positive control. Its removal 2 hpi did not impact viral replication to levels comparable to DMSO treatment, suggesting that cherylline targets a viral process occurring after that time point (Fig. 5A). Similarly, cherylline removal 2 hpi restored virus replication completely (Fig. 5A), as did removal of hippadine, flexinine, gigantellinine, sanguinine, and crinine (Fig. S3B). Restoration of infection upon removal confirmed that cherylline is not virucidal and that it inhibits a step downstream viral entry. Following viral endocytosis, initiation of translation of viral RNA and protein maturation begins as early as 1 hpi. RNA synthesis follows 6 hpi, and virion assembly, maturation, and exocytosis arise from 12 hpi. We tested the impact of cherylline addition at 0, 2, 4, 7, 12, and 24 hpi on the percentage of DENV_GFP_-infected Huh7 cells 72 hpi. As expected, the RNA synthesis inhibitor NITD008 abrogated GFP expression when added at 0, 2, 4, 7, or 12 hpi, prior to or during RNA synthesis (Fig. 5C). Low levels of infection rose when NITD008 was added at 24 hpi, confirming its activity at prior timing of the DENV life cycle, during RNA replication. Cherylline entirely blocked viral replication when added between 0 and 7hpi (Fig. 5C), supporting the idea that it acts at a postentry step of the viral cycle that occurs from/after 7 hpi. At 12 hpi, some cherylline antiviral activity was lost, suggesting that it optimally acts before that time of the life cycle. When added at 24 hpi, cherylline lost one-third of its inhibitory potential, consistent with an activity during the first infection. From our previous experiment (Fig. 5B), we excluded an effect on virion infectivity and viral entry, while these results suggest that cherylline possibly targets either translation, protein maturation, or RNA replication.

Next, cherylline was added to the cells at the time of infection and then removed at each indicated time point (Fig. 5C and D). After removal of compounds, successfully produced DENV infectious particles replicate further until the time of analysis, amplifying overall infection levels. As expected, the effect of the NS5 inhibitor NITD008 was lost when the drug was removed at 2 and 4 hpi, before RNA synthesis initiation. The continuing presence of the drug until 7 hpi was necessary to its the antiviral activity. Cherylline-dependent inhibition of viral replication was lost when the AA was removed at 2, 4, or 7 hpi, while its presence for the first 12 h of the viral life cycle successfully impaired DENV replication. Similar kinetics of inhibition were observed for the other AAs tested (Fig. S4D and E). Altogether, these results indicate that cherylline acts during the RNA synthesis of the DENV life cycle. This is further supported by the fact the profile of cherylline inhibition kinetics closely mirrors that of NITD008, a potent flaviviral polymerase inhibitor.

We next challenged this mode-of-action model in an experimental setup in which the viral RNA genomes replicate without the entry and assembly steps. We took advantage of DENV subgenomic (sg) replicons (sg-DVs-R2A WT) and RNA genomes that are defective in RNA replication (sg-DVs-R2A GND, mutated GDD motif in NS5 RNA-dependent RNA polymerase [RdRp]) (Fig. 5E). None of these genomes express structural proteins in transfected cells; hence, no viral particles are produced, and virus release or entry does not occur. Sg-DVs-R2A WT RNA replicates and is translated like the WT full-length genome; luciferase emission is directly proportional to the efficacy of viral RNA replication and translation. *In vitro* transcribed sg-DVs-R2A WT RNA was transfected into Huh7.5 cells and treated with DMSO, NITD008, or cherylline. At 48 to 72 h postelectroporation (hpe), both compounds were efficient in blocking viral RNA replication (3 log_10_ reduction of relative light units [RLU]) during steps that occurred after entry and before release (Fig. 5F; S4C). In contrast, sg-DVs-R2A GND does not replicate (Fig. S4F), and hence, luciferase activity 4 hpe reflects the efficacy of DENV RNA translation (Fig. 5E). Transfection using the RdRp-deficient replicon led to a very different profile. In control DMSO-treated cells, luciferase luminescence was high at 4 hpe and waned significantly at 24 h, in accordance with the absence of replication of this system (Fig. S4G). Cherylline, like NITD008, had no effect on the luminescence of this genome at 4 hpe, demonstrating that they do not interfere with protein synthesis (Fig. 5G). Altogether, these results unambiguously demonstrate that cherylline disrupts the RNA synthesis step of the DENV life cycle and not its infectivity, entry, and translation.

### *In silico* reverse screening of cherylline targets.

Cherylline has been poorly studied, and its cellular and viral targets are unknown. We used *in silico* algorithms to get more insight into cherylline’s possible mode of action. Shape- and pharmacophore-based reverse screening with PharmMapper, ChemMapper, SwissSimilarity, and SwissTarget did not uncover any viral protein as hits, indicating that cherylline is not homologous to any currently known viral inhibitors included in the databases that were screened. Several human proteins, such as dopamine and estrogen receptors, as well as neurotransmitter transporters, and cell division protein kinase 5 were predicted to interact with cherylline (Tables S1 and S2), suggesting that it might target cellular proteins implicated in the viral life cycle. Interestingly, estrogen and neurotransmitter-interacting proteins were most often uncovered as hits. Finally, we used SwissADME to calculate ADME (absorption, distribution, metabolism, and excretion) and Lipinski’s parameters of cherylline (Table S3) and other AAs. Cherylline structure respects the five rules of Lipinski with a molecular weight of <500 Da, a liposolubility (octanol/water) logP of <4.15, fewer than 10 electron acceptors and fewer than 5 donors, and a biodisponibility score of 0.55. This prediction suggests that cherylline possesses the chemical properties that are compatible with therapeutic usage in humans.

### Cherylline inhibits DENV infection in peripheral blood mononuclear cells.

**FIG 6:**
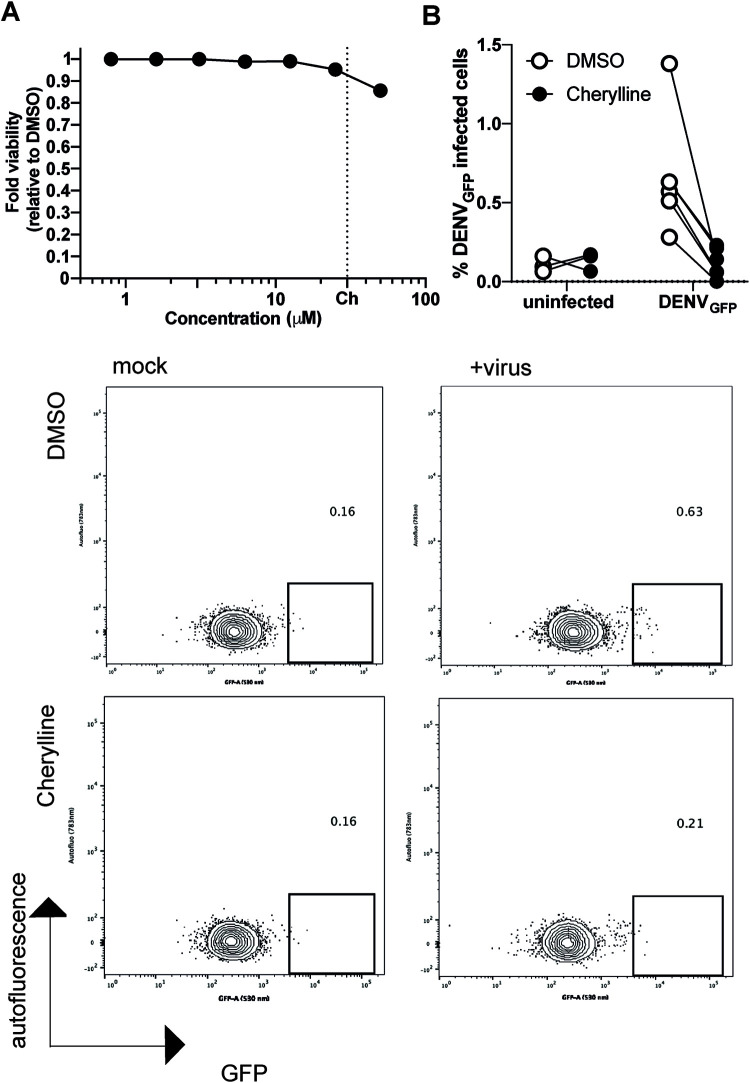
Cytotoxicity and antiviral effects of cherylline in PBMCs. (A) The cytotoxicity of antiviral concentrations of cherylline in PBMCs was measured by ATP production and normalized over DMSO at 48 h posttreatment. Means with SEM are shown. Dotted lines represent the concentration used in the antiviral assay. Ch, cherylline. (B) Antiviral activity of cherylline in PBMCs. PBMCs were infected with DENV_GFP_ preincubated with anti-Envelope antibody 4G2 at an MOI of 2, treated with cherylline (30 μM) or DMSO (0.05%), and analyzed 72 hpi by flow cytometry. The experiment was performed in triplicates (uninfected) and six-plicates (infected). Representative plots are shown.

Finally, we wanted to validate cherylline antiflaviviral potential in a human primary cell model relevant to dengue disease, namely, peripheral blood mononuclear cells (PBMCs) (38). PBMCs were infected with DENV_GFP_ particles which were preincubated with a panflaviviral antienvelope antibody to increase infection levels in monocytes through antibody-dependent enhancement (39, 40). Infected PBMCs were then treated for 72 h with 30 μM cherylline, a concentration that was not toxic for these primary cells (Fig. 6A). Of note, NITD008 was excluded from further analysis because it was cytotoxic in PBMCs. The percentage of infected cells (GFP-positive cells) was determined using flow cytometry (Fig. 6B). In DMSO-treated control cells, GFP intensity was low but readily detectable, and a median of 0.6% GFP^+^ cells were productively infected with DENV_GFP_. Cherylline treatment led to a 5.3-fold decrease in the percentage of GFP^+^ cells (*P = *0.0011, Mann-Whitney test), with a median of 0.1% GFP^+^ cells, similar to background levels (Fig. 6C). These results validate the anti-DENV potential of cherylline in human primary blood cells, a target of DENV during pathogenesis.

## DISCUSSION

In a previous study, we showed that several alkaloids from *C. jagus* display cytotoxicity and anticholinesterase activity (24). Here, we uncovered that crude alkaloid extract from *Crinum jagus* inhibited DENV infection *in cellulo*. We further investigated the antiviral potential of the 9 alkaloids of crinine, lycorine, galanthamine, and cherylline ring types isolated from this plant. While lycorine’s antiviral activity is well known, the potential of other AAs has not been previously characterized. Here, we investigated their activity on DENV and ZIKV and HIV-1 infection.

First, we discovered that in addition to lycorine, *C. jagus* extract contains another compound that efficiently blocks DENV, namely, cherylline. Other AAs isolated from *C. jagus* displayed weak activity at high concentrations (gigantelline, gigantellinine, and crinine- and galanthamine-type structures) or were highly cytotoxic (hippadine).

We confirmed the anti-DENV activity of cherylline, measuring the inhibition of viral replication through modulation of the Rluc reporter gene expression. Because of the genetic proximity between DENV and ZIKV proteins, we tested cherylline anti-ZIKV activity and determined that it also efficiently blocked ZIKV replication. Then, to gain understanding of the specificity of cherylline’s antiviral activity, we tested its effect on HIV-1 using a replication-deficient virus pseudotyped with a vesicular stomatitis virus G (VSV-G) envelope. Thus, if entry, assembly, or release were targeted by tested compounds, this would be missed by the current assay. We did not detect any antiretroviral activity from cherylline or from any of the AAs tested against this virus. Lycorine was reported to inhibit HIV-1 infection in some studies (41, 42), while others have demonstrated that it was a very poor inhibitor of HIV-1 reverse transcription (RT) (43) or that it did not inhibit HIV-1 (26). Its cytotoxicity could be responsible for these discrepancies, as even at antiviral concentrations (1 to 5 μM), lycorine treatment nonspecifically increases the detected antiviral activity because of the progressive depletion of viable permissive cells. Nonetheless, our results unequivocally show that HIV-1 life cycle steps between entry and release are not targeted by the studied AAs in THP-1 cells. Interestingly, a study published in 1989 reported that cherylline derivatives had no significant effect against the DNA virus herpes simplex virus (44). Altogether, this suggests that cherylline antiviral activity exhibits some selectivity to flaviviruses.

Then, we assessed the effect of cherylline on the replication of WT strains of ZIKV and DENV2. Cherylline dampened viral titers from the pathogenic ZIKV H/PF/2013 strain about a 100-fold. It diminished replication of WT ZIKV MR766 and WT DENV2 16881s 8- and 17-fold, respectively, to very low levels compared to the control. These experiments were performed in the highly permissive Huh7.5 and Vero cell lines, which are more permissive than Huh7 cells to flaviviral replication. Hippadine hindered French Polynesian ZIKV replication, albeit at concentrations that displayed cytotoxicity. It had little effect on the propagation of other strains of ZIKV and on WT DENV-2. Gigantellinine and flexinine had little effect on all three viral strains.

Having established that cherylline inhibited flavivirus infection, we investigated the life cycle steps that could be targeted. We removed the drug 2 hpi, which abolished its antiviral effect, showing that cherylline was not viricidal and that it did not prevent binding or viral entry into cells. We pursued identification of the viral step that was targeted using time-of-drug-addition and -removal assays. Cherylline treatment did severely impede viral replication when added between 0 and 7 hpi, whereas its antiviral potency was lost when removed at 2, 4, or 7 hpi and recovered at 12 hpi. Thus, its presence between 7 and 12 hpi is required to successfully impair infection, suggesting that it acts at a postentry step, possibly during RNA synthesis. By using subgenomic replicons and replication-deficient genomes, we confirmed that cherylline did inhibit the RNA replication step of the DENV life cycle but did not target infectivity, entry, or translation. We used a hygromycin-resistant replicon to attempt to isolate escape mutants and identify cherylline’s potential viral target. Unfortunately, cells containing resistant DENV subgenomes were not generated following a continuous treatment of 30 days. This does not exclude a direct effect of cherylline on viral enzymes such as RdRp, as similar observations were made using the NITD008 nucleoside analog (35). Instead, these results support the finding that cherylline exhibits a strong barrier to resistance.

The replication complex of DENV virus in the endoplasmic reticulum is composed of its nonstructural proteins in interaction with several cellular proteins, all playing a key role in viral replication (45–48). Lycorine also inhibits the RNA replication step, but the exact mechanism is not known. Chen et al. showed that high concentrations of lycorine (100 μM) inhibited 80% of ZIKV NS5 RdRp activity *in vitro* (28), but this concentration is 100-fold higher than what is tolerated by cells. Others reported that lycorine had no effect on West Nile virus NS5, but rather, that its activity was dependent on the valine at the 9th position of the 2K peptide (27). The 2K peptide is part of the replication complex, playing a key role in polyprotein maturation and topology as well as in replication organelle biogenesis (49–52). Lycorine and cherylline structures contain distinct ring types. Although AAs are known for their pleiotropic effects, cherylline does not display the same broad antiviral spectrum as lycorine. Cherylline targeted multiple proteins to achieve its antiviral activity. It interacted with human proteins rather than viral proteins, such as dopamine and estrogen receptors, as predicted by *in silico* reverse screening. Indeed, the dopamine receptor D1 and D4 antagonists prochlorperazine and dihydrobenzothiepines have been described to inhibit DENV replication (53, 54), but they target early steps of DENV replication, i.e., viral binding and entry, in contrast to cherylline. Recently, estrogen receptor modulators were also identified as flavivirus inhibitors, independently of the estrogen receptor itself. Cyclofenil, which interacts with NS1, and raloxifene both target RNA replication and polyprotein translation by an unknown mechanism (55, 56). Cyclofenil also impacted viral assembly/maturation (56). Although these inhibitors do not share the same mode of action as cherylline, this emphasizes that many cellular or viral proteins could be targeted by the AA. In any case, further investigations are required to uncover the target, which will enable interaction studies and, potentially, optimization and identification of resistance mechanism.

Despite their strong similitude in structure with cherylline (Fig. 2A), gigantelline and gigantellinine effects were much poorer. These disparities are probably due to distinct functionalization or stereochemistry of their shared carbon skeleton. As cherylline and gigantellinine hold the same stereochemistry, the distinction between the two compounds associated with the reduction of antiviral potential, rather, lies on the presence of a further hydroxyl group at C-5′ and/or methyoxylation of the hydroxyl group at C-4′ in the C-ring of gigantellinine. In the case of gigantelline, the further loss of activity is associated with a singular stereochemistry of the junction between B/C rings and/or to the methoxylation of the hydroxyl group at C-7 of its A ring. Palmatine, another isoquinoline alkaloid, was shown to display antiflaviviral potential, although higher concentrations were required to inhibit DENV with higher cytotoxicity (EC_50_, 26.4 μM and CC_50_, 1,031 μM) (57). Plamatine’s structure is distinct from cherylline’s in several aspects, highlighting that the *para*-substituted phenol ring could play a role in the observed differences in antiviral activity. Thus, our study provides some insight into cherylline structural determinants required for its antiflaviviral activity, which will have to be taken into consideration for chemical optimization in future structure-activity relationship studies.

Finally, we validated cherylline antiviral activity in human primary blood cells that are targeted by DENV in the natural course of infection *in vivo*. PBMCs are generally poorly permissive *in vitro* (58, 59), but they include monocytes, which are believed to be important targets of DENV and to contribute to viral dissemination in patients (60, 61). Cherylline was not cytotoxic to PBMCs and efficiently inhibited DENV_GFP_ infection in these cells. Importantly, lycorine has been successfully used in mice to fight ZIKV (28), highlighting that AAs may provide therapeutic gain. Although cherylline can be extracted from several Amaryllidaceae species, mostly from the *Crinum* genus (62–64), it is considered a rare alkaloid. Fortunately, cherylline chemical synthesis has been successfully performed through several methods (65–67), as reviewed in reference 68, providing an alternative method of production. AA’s biosynthesis pathway is beginning to be uncovered (15); once the enzymes responsible for cherylline synthesis are confirmed, exogenous introduction of encoding genes into yeasts or microalgae will be an interesting strategy to produce AAs.

### Conclusion.

Until now, cherylline was mostly known for its moderate antiacetylcholinesterase activity and has never been associated with antiflaviviral activity before, nor have any derivatives. We show that most AAs from *Crinum jagus* display a low to moderate effect against DENV. Our study shows that cherylline inhibits replication of both DENV and ZIKV. Although the EC_50_ obtained for cherylline was quite high, its low cytotoxicity in PBMCs makes it an interesting lead compound to fight DENV and ZIKV and could pave the way to new therapeutic strategies.

## MATERIALS AND METHODS

### *Crinum jagus* crude extract and GC-MS analysis.

Bulbs of *C. jagus* (*giganteum*) were collected in Senegal in the Montrolland district (14°55′56.22″N and 16°59′38.62″W) in 2018 and taxonomically identified by a senior scientist from the Herbarium of IFAN of the University Cheikh Anta Diop in Dakar. Alkaloids were extracted from dried bulbs of *C. jagus* using the method described in reference 69. High-performance liquid chromatography with a diode-array detector (HPLC-DAD) and thin-layer chromatography (TLC) confirmed the presence of alkaloids in the extract. Gas chromatography-mass spectrometry (GC-MS) analysis was performed using the method described in reference 70. Alkaloids were identified by comparison with the National Institute of Standards and Technology (NIST 05) database based on matching mass spectra. The total ion current (TIC) percentage provided in Table 2 was connotated with the proportion of each compound in the extract. The area under the GC-MS peaks depends both on the concentration of the related compounds and on the intensity of their mass spectrum.

**TABLE 2:** Alkaloids identified by GC-MS in *C. jagus* bulb extract^*a*^

Alkaloid	[M^+^]	BP	RT (min)	CJ (%)	Identification
Vittatine/crinine	271	271	21.871	24.418	NIST 05 Database
Cherylline	285	242	23.173	9.413	NIST 05 Database
Lycorine	287	226	25.871	19.68	NIST 05 Database
Unidentified	299	225	23.531	31.422	NA
Unidentified	315	241	25.030	15.68	NA

a Values are expressed as percentages of total ion current (TIC) for the relative quantitative CJ. BP, base peak; RT, retention time (in minutes); CJ, *C. jagus*; NA, not applicable.

### Amaryllidaceae alkaloids.

Lycorine, hippadine, sanguinine, crinine, gigancrinine, flexinine, cherylline, gigantelline, and gigantellinine were isolated from *C. jagus* as reported in reference 24. Their purity, >98%, was ascertained by ^1^H NMR and liquid chromatography mass spectrometry (LC/MS) spectrometry. Each AA was dissolved in dimethyl sulfoxide (DMSO) at 100 mM as a stock solution and stored at –20°C until further usage. At the time of use, they were diluted in Dulbecco’s modified Eagle’s medium high glucose (DMEM) or RPMI medium to the desired concentration. DMSO was used as a negative control, whereas guanosine analogue ribavirin and flaviviral NS5 inhibitor NITD008 (Tocris Bioscience) were used as positive antiviral controls.

### Cell culture.

The human hepatocarcinoma Huh7 cell line was kindly provided by Hugo Soudeyns. Murine LL171 reporter cells (71), Crandell-Rees Feline Kidney (CRFK), and Huh7 cells were maintained in DMEM supplemented with 10% fetal bovine serum (FBS) and 1% penicillin-streptomycin (PS) solution (all from Wisent, Inc., Canada). Hepatocarcinoma RIG-I-deficient Huh7.5 (a kind gift from Patrick Labonté), and Vero E6 (kindly shared by Tom Hobman) cells were cultured in DMEM (Life Technologies) containing 10% FBS, 1% PS, and 1% nonessential amino acids (Thermo Fisher). Human embryonic kidney HEK293T and human monocytic cell line THP-1 were grown in RPMI (Wisent) with 10% FBS and 1% PS. Frozen human peripheral blood mononuclear cells (PBMCs) were obtained using Institutional Review Board (IRB)-approved consent forms and protocols (Stem Cell Technologies). They were thawed in 20% FBS containing RPMI prewarmed at 37°C, washed twice, and incubated for 6 h. Medium was replaced by RPMI with 10% FBS and 1% PS before cytotoxic and antiviral assays. All cells were maintained at 37°C and 5% CO_2_.

### Cytoxicity assay.

Cytotoxicity properties were titrated by metabolism monitoring using the XTT [2,3-bis-(2-methoxy-4-nitro-5-sulfophenyl)-2*H*-tetrazolium-5-carboxanilide] assay kit (Roche, Millipore Sigma), as described before (24). Briefly, Huh7 cells were seeded at 10 × 10^3^ cells per well in 96-well plates. Concentrations of alkaloid bulb extract ranging from 0.019 to 2.5 μg/ml were added the next day. After 72 h of incubation, medium was replaced by phenol-free DMEM (Wisent) containing 0.3 mg/ml XTT, and cells were incubated for 4 h. Absorbance was measured at 450nm using a microplate spectrophotometer (Synergy H1, Biotek). Treatment conditions with 0.5% DMSO were used as the reference control. The percentage of cell viability was calculated at each concentration.

Where indicated for Huh7.5 and Vero E6 cells and PBMCs, cell viability was measured by monitoring of cellular ATP levels using the Cell-Titer GLO assay kit (Promega). Briefly, 10× 10^3^ to 20 × 10^3^ cells in 50 to 100 μl were seeded in 48-well or 96-well plates and cultured overnight. AAs were added at the designated concentrations for 24 or 48 h, as specified in the text. Then, 50 to 100 μl of room temperature Cell-Titer GLO reagent was added in each well to room temperature-equilibrated cell plates. Plates were rocked for 2 min and rested for 10 min, and the luminescence signal was measured using a microplate spectrophotometer (Biotek Synergy H1 or Tecan Spark multimode microplate reader). Viability was expressed as a fold change calculated using the value of DMSO-treated cells at each concentration; for each AA and CC_50_, values were determined.

### Preparation of flaviviral genomes.

Plasmids for DENV or ZIKV vectors which encode GFP (pFK-DVs-G2A for DENV_GFP_), Renilla luciferase (pFK-DVs-R2A, serotype 2, strain 16681 for DENV_R2A_; pFL-ZIKV-R2A strain FSS13025 for ZIKV_R2A_) reporter genomes, wild-type (WT) DENV (pFK-DVs; 16681), subgenomic (sg) replication-deficient NS5 mutant (sg-DVs-R2A-GND), and WT (sg-DVs-R2A-WT) subgenomic replicon systems were all obtained from Ralf Bartenschlager and Pei-Yong Shi. Plasmids were linearized with XbaI (DENV) or ClaI (ZIKV) and purified, and 1 μg was *in vitro* transcribed using the mMESSAGE mMACHINE T7 or SP6 transcription kit (Invitrogen). The resulting RNA quality and concentration were assessed by migration on a 0.8% agarose gel and Nanodrop spectrometry (Thermo Scientific), respectively.

### Production of DENV and ZIKV stocks.

Subconfluent trypsinized Vero E6 cells were washed once and resuspended in cytomix buffer (120 mM KCl, 0.15 mM CaCl_2_, 10 mM potassium phosphate buffer, 25 mM HEPES, 2 mM EGTA, 5 mM MgCl_2_ [pH 7.6], freshly supplemented with 2 mM ATP and 5 mM glutathione) at a density 1.5 × 10^7^ cells/ml, as described in reference 45. Then, 10 μg of *in vitro*-transcribed viral RNA genomes (from pFK-DVs, pFK-DVs-R2A, pFK-DVs-GFP, pFL-ZIKV, and pFL-ZIKV-R2A) were mixed with 400 μl of cells, transferred into an electroporation cuvette (Bio-Rad; 0.4-cm gap width), and pulsed once with a GenePulser Xcell instrument (Bio-Rad) at 975 μF and 270 V. Cells were immediately transferred to prewarmed complete DMEM and seeded. Culture medium was changed 24 h postelectroporation (hpe). Virus-containing cell culture supernatants were harvested 4 to 8- days postelectroporation, concomitantly with the appearance of cytopathic effect (CPE). Virus stocks were filtered through 0.45-μm syringe filters and supplemented with 10 mM HEPES (pH 7.5), and aliquots were stored at –80°C until use. ZIKV H/PF/2013 (French Polynesia) and ZIKV MR766 (Uganda) virus stocks were obtained from the European Virus Archive Global (EVAg). ZIKV stocks (ZIKV H/PF/2013 and ZIKV MR766) were amplified and produced in Vero E6 cells. Plaque assays (described below) were used to determine the infectious titers of the virus stocks (72).

### AA anti-ZIKV and -DENV activity.

Briefly, Huh7 cells were seeded at 15 × 10^3^ cells per well in 48-well plates and cultured for 16 h. Cells were pretreated with the indicated concentrations of *C. jagus* bulb crude extract or AAs for 2 h and infected with DENV_GFP_ at an MOI of 0.1 to 0.3, depending on the assay, and further incubated for 72 h at 37°C. PBMCs were infected with DENV_GFP_ (MOI, 2) preincubated with the panflaviviral antienvelope 4G2 antibody (clone D1-4G2-4-15; Sigma-Aldrich) for 30 min at 4°C to enhance infection through antibody-dependent enhancement (73, 74). Cells were washed 24 hpi, and further incubated for 48 h. GFP signal of infected cells was visualized on an Axio Observer microscope (Carl Zeiss, Inc., Toronto, ON, Canada) or measured on a flow cytometer (FC500 MPL cytometer or a BD FACSMelody; BD Lifesciences-Biosciences) and analyzed with FCS express 6 and FlowJo software (BD).

To determine the half-maximal effective concentration (EC_50_), Huh7.5 cells were plated in 10-mm petri dishes and infected with DENV_R2A_ and ZIKV_R2A_ (MOI, 0.005). Virus inoculum was removed 2 hpi, and cells were washed with PBS, trypsinized, and plated in a 96-well plate with various concentrations of AAs. After 48 h, luciferase production was monitored as described below.

Subgenome RNAs (sg-DVs-R2A-GND, sg-DVs-R2A-WT, and sg-DVs-R2H) were directly transfected into Huh7.5 cells by electroporation, and luminescence was measured at the indicated time points following the procedures described below.

### Luciferase detection.

Luminescence emitted from virus-encoded Renilla luciferase (Rluc) was measured to monitor viral replication (DENV-R2A, sg-DVs-R2A-WT, and ZIKV-R2A) or viral protein translation (sg-DVs-R2A-GND). Infected cells were lysed in 100 μl lysis buffer (0.1% Triton X-100, 25 mM glycylglycine [pH 7.8], 15 mM MgSO4, 4 mM EGTA [pH 8], and 1 mM DTT). Rluc assays was performed by injecting 150 μl of assay buffer (25 mM glycylglycine [pH 7.8], 15 mM MgSO4, 4 mM EGTA [pH 8], and 15 mM K2PO4 [pH7.8]) and coelenterazine (1.43 μM Prolume) to 30 μl of the lysate, as described in reference 75. Luminescence was measured in a Spark multimode microplate reader (Tecan).

### RNA extraction and RT-qPCR.

TRIzol reagent (Thermo Fisher Scientific) and chloroform (Sigma-Aldrich) (for experiments using DENV_GFP_) or an RNeasy kit (Qiagen; for experiments using WT DENV and ZIKV) were used to extract total RNA from infected and control cells at 12 and 24 h postinfection according to the manufacturers protocol. Glycogen (Thermo Fisher Scientific) was added during the extraction. Cell lines, virus types, and MOI are specified in the figure legends for each experiment. Viral RNA levels were assessed through a one-step reverse transcriptase quantitative PCR (RT-qPCR) protocol with a One Step TB Green PrimeScript RT-PCR kit II (perfect real time) (TaKaRa Bio, Inc.) in the case of DENV_GFP_. Amplification and fluorescence detection were performed using 2 μl template (250 to 500 ng) and 400 nM forward and reverse primers in 20 μl final volume on an Mx3000 real-time PCR system (Agilent). A reverse transcription step was performed at 42°C for 5 min and 95°C for 10 sec, and PCR was performed for 40 cycles at 95°C and 60°C for 34 s, followed by a dissociation curve from 60 to 95°C. Relative viral RNA abundance was normalized to GAPDH mRNA levels. The threshold cycle (*C_T_*) was determined using MxPro software (Agilent). In the case of WT viruses, reverse transcription was performed with 800-ng template RNA using SuperScript IV VILO master mix with an ezDNase enzyme kit (Invitrogen). Amplification and fluorescence detection were performed using PowerUP SYBR green master mix (Applied Biosystems), 5 μl cDNA, and 300 nM forward and reverse primers in 10 μl final volume on a LightCycler 96 device (Roche). A reverse transcription step was performed at 25°C for 10 min, 50°C for 10 min, and 85°C for 5 min. qPCR was performed at 50°C for 2 min, 95°C for 2 min, and then 40 cycles of 95°C for 15 sec and 60°C for 1 min. Primers sequences were as follow: (i) for DENV 16681s, 5′-GCCCTTCTGTTCACACCATT-3′ and 5′-CCACATTTGGGCGTAAGACT-3′; (ii) for ZIKV H/PF/2013, 5′-AGATGAACTGATGGCCGGGC-3′ and 5′-AGGTCCCTTCTGTGGAAATA-3′; and (iii) for GAPDH, 5′-GAAGGTGAAGGTCGGAGTC-3′ and 5′-GAAGATGGTGATGGGATTTC-3′. Relative expression was calculated using the ΔΔ*CT* method with GAPDH for normalization and normalized on background levels in noninfected cells.

### DENV and ZIKV titration by plaque assay.

Huh7.5 cells were infected with DENV 16881s, ZIKV H/PF/2013, or ZIKV MR766 (MOI, 0.1) or left uninfected. Viral inoculum was removed 2 hpi, and cells were treated with AAs, NITD008, or DMSO (vehicle). Supernatants were harvested 2 days pi, and PFU were determined by plaque assay on VeroE6 cells. Cells incubated overnight (2 × 10^5^ cells/well in 24-well plates) were infected with 10-fold serially diluted virus-containing supernatants for 2 h at 37°C. Inoculum was then replaced with MEM (Life Technologies) containing 1.5% carboxymethylcellulose (Millipore-Sigma). ZIKV- and DENV-infected cells were incubated for 5 and 7 days, respectively. One volume of 10% formaldehyde was added to the cells for fixation. Two hours later, cells were washed with tap water and stained with a 1% crystal violet/10% ethanol solution for 30 min. Wells were rinsed with tap water, and the number of plaques were counted to calculate virus titers.

### Time-of-drug-addition and -removal assays.

Huh7 cells (1.5 × 10^4^ per well) were seeded in 48-well plates and infected with DENV_GFP_ at an MOI of 0.15. For the time-of-drug-addition assay, at the time of infection, AAs were added in wells corresponding to 0 h. At 2 hpi, viral inoculum was removed from every well, and AAs were added back to wells corresponding to 0 h and 2 h. At 4, 7, 12, and 24 hpi, AAs or DMSO diluted in DMEM was added to the appropriate wells. After 72 h, cells were trypsinized and fixed in 4% formaldehyde to measure the percentage of infected cells on an FC500 MPL cytometer. Alternatively, pictures of Hoechst 33342-stained cells were acquired on an Axio Observer microscope. All assays were performed in triplicate.

For the time-of-drug-removal assay, Huh7 cells were seeded in 48-wells plates (1.5 × 10^4^ cells per well), infected with DENV_GFP_ at an MOI of 0.15, and treated with medium containing AAs at the indicated concentrations at the time of infection. At 2 hpi, plates were washed, and medium was replaced to remove remaining viruses in the supernatant. AAs were added back to all wells, except the ones corresponding to 2 h of treatment, in which fresh medium exclusive of any AA was added. Accordingly, after 4, 7, and 12 h of infection, the supernatant was replaced by fresh medium in corresponding wells. After 72 h of incubation, results were acquired as for the time-of-drug-addition assay. All assays were performed in triplicate.

### Production of VSV-G-pseudotyped human immunodeficiency virus (HIV)-1 vectors.

pNL4-3_GFPΔEnvΔNef_ is replication-incompetent due to a deletion that produces a frameshift in *env*, and *nef* is replaced by *gfp* (green fluorescent protein) as described in reference 76. pNL4-3_GFPΔEnvΔNef_ and pMD.G (a plasmid encoding vesicular stomatitis virus G glycoprotein [VSV-G]) were prepared from bacterial stocks using the Qiagen MidiPrep kit. To produce HIV-1_GFP_ vectors, plasmids were cotransfected into 90% confluent HEK293T cells in 10-cm culture dishes using polyethylenimine (PEI; Polysciences, Niles, IL) as described in reference 77. Medium was changed 6 to 16 h posttransfection. Supernatants containing HIV-1_GFP_ were harvested 24 h later, centrifuged for 10 min at 3,000 rpm, 0.45-μm-filtered, and stored at –80°C. The MOI was assessed by measuring the infectivity of serially diluted vector preparation in CRFK cells.

### HIV-1_GFP_ infectivity assay.

AA antiretroviral activity was evaluated using HIV-1_GFP_ in THP-1 cells. Briefly, THP-1 cells were seeded at 1.5 × 10^4^ cells per well in 96 well-plates and incubated overnight. Cells were pretreated with two concentrations of AAs for 2 h and then infected with HIV_GFP_ at an MOI of 1. After 72 h, the percentage of infected cells was measured using an FC500 MPL cytometer (Beckman Coulter, Inc., California) and analyzed using FCS Express 6 software (De Novo Software, California). DMSO and nevirapine (Sigma-Aldrich, Canada) were used as a negative and a positive control, respectively. All assays were performed in triplicate.

### Type I IFN activation assay.

*In vitro* type I IFN activation was measured in LL171 cells using the luciferase assay system kit (Promega). Briefly, 200 μl LL171 reporter cells (L929 cells expressing an IFN stimulated response element [ISRE]-luciferase [78]) were seeded at 1.5 × 10^4^ cells/well in 96-well plates and cultured for 16 h. Medium was replaced with DMEM containing cherylline for 24 h. Supernatant was removed, cells were rinsed with PBS, and lysis buffer was added (luciferase assay reagent; Promega). Then, cells were scraped and transferred into opaque 96-well plates. LAR (luciferase assay reagent; Promega) was added to each well, and luminescence was measured at 480 nm using a microplate spectrophotometer (Synergy H1). 5,6-dimethylxanthenone-4-acetic acid (DMXAA, 20 μg/ml) was used as a positive control. All assays were performed in triplicate.

### *In silico* characterization of cherylline.

SwissSimilarity was used to screen analogous compounds in the PDB database (79). SwissTargetPrediction (80), ChemMapper (81), and PharmMapper (82) were used for the virtual reverse screening of cherylline’s possible targets. SwissADME (absorption, distribution, metabolism, and excretion) was used to predict the ADME properties of the cherylline drug (83).

### Statistical analyses.

Graphs and statistical analyses (EC_50_, CC_50_) were performed with GraphPad Prism 7 software. The nonparametric Mann-Whitney test was used, and *P* values of ≤0.05 were considered significant.
