# Supplementary material for: Amaryllidaceae Alkaloid Cherylline Inhibits the Replication of Dengue and Zika Viruses
Source: Antimicrob Agents Chemother. 2021 Aug 17;65(9):e00398-21. doi: 10.1128/AAC.00398-21 (PMC8370201; doi:10.1128/AAC.00398-21)
Supplement: Supplemental file 1 — Supplemental material. Download AAC.00398-21-s0001.pdf, PDF file, 0.9 MB [file aac.00398-21-s0001.pdf]

Supplemental Files

Supplementary Figure S1.

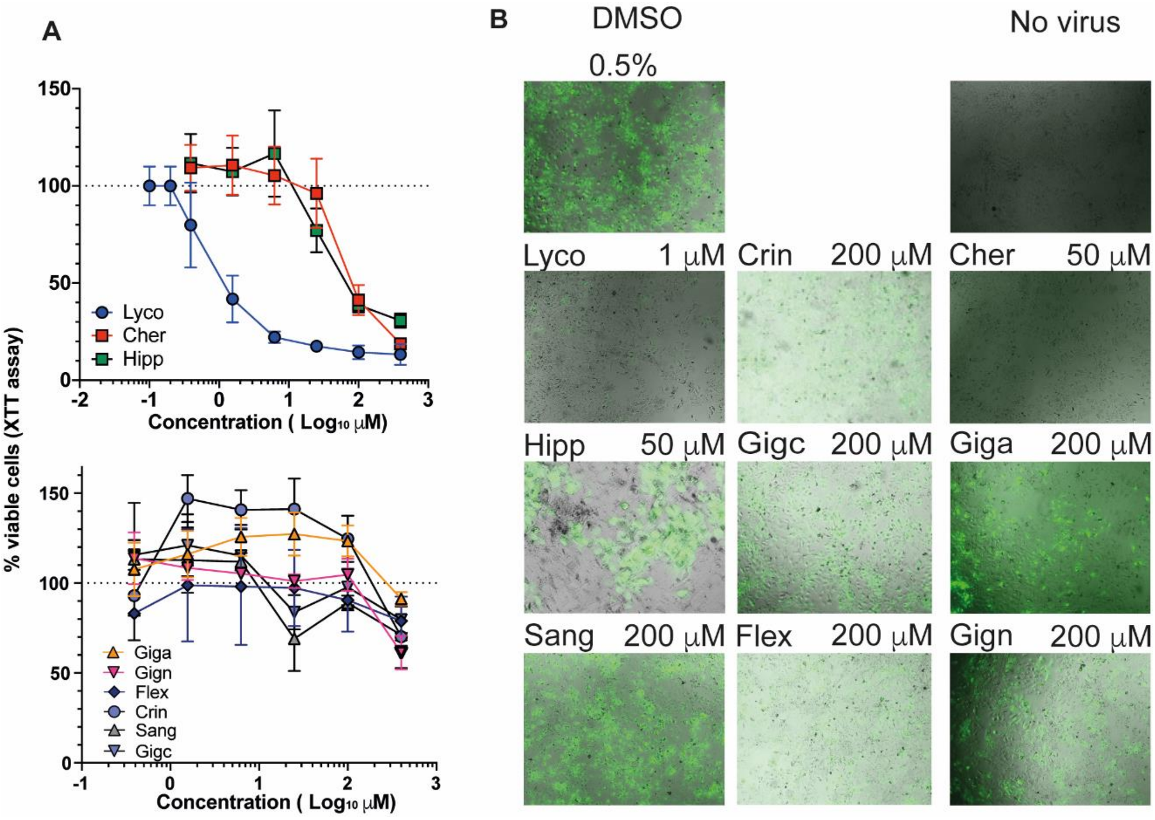

Fig. S1. Cytotoxicity of alkaloids and $DENV_{GFP}$ infection. A. Cytotoxic activity of AAs was assessed at concentrations ranging from 0.4 to 400 μM after incubation for 72 hours on Huh7 cells with an XTT assay. Experiment was performed in triplicates. The % of viable cells was determined by performing a ratio of luminescence for untreated vs treated cells at 450 nm, x 100. Upper graph shows cytotoxic alkaloids lycorine, hippadine and cherylline, while lower graph displays weakly cytotoxic alkaloids. B. Merged pictures of brightfield and GFP channels of Huh7 cells treated with alkaloids and infected with $DENV_{GFP}$, corresponding to pictures of Fig. 2B.

Supplementary Figure S2.

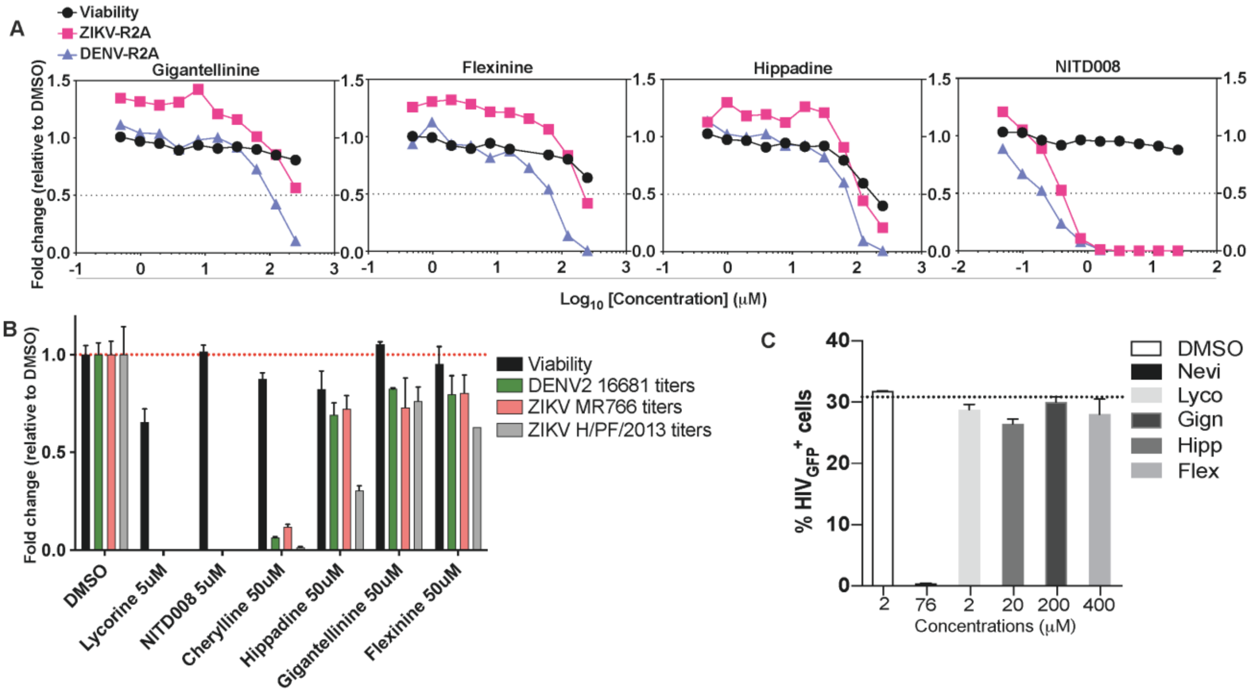

Fig. S2. Screening of hippadine, gigantellinine and flexinine activity against DENV and ZIKV replication. A. Antiviral activity of hippadine, gigantellinine and flexinine was studied by measuring the levels of $DENV_{R2A}$ (purple) and $ZIKV_{R2A}$ (pink) replication in Huh7.5 cells through luciferase luminescence at 72 h p.i.. Cell viablity (ATP) was assessed at 24 h. NITD008 was used as a positive control. Results are displayed as fold changes in viability and replication, 1 meaning no change compared to DMSO-treated cells. B. Viral titers were measured by plaque assay on Vero E6 cells using cytopathic WT French Polynesia H/PF/2013 and Uganda MR766 ZIKV and DENV2 16881s strains. Huh7.5 cells were infected and treated 2 h p.i. with compounds. 48 h later, supernatants were harvested and plated on Vero E6 cells. Fold replication was calculated from viral titers values. Viability (ATP) was calculated at 48 h p.i. C. THP-1 cells were pre-treated with lycorine (lyco), hippadine (hipp), gigantellinine (gign) and flexinine (flex) in triplicates for 2 h, and infected with VSV-G pseudotyped HIV-1_{GFP} at MOI=1. The antiretroviral nevirapine (nevi) was used as a positive control, while 2% DMSO was used as a negative control. Results were analyzed by flow cytometry 72 h p.i.. Experiment was performed in triplicates with two concentrations, the highest being shown.

Supplementary Figure S3.

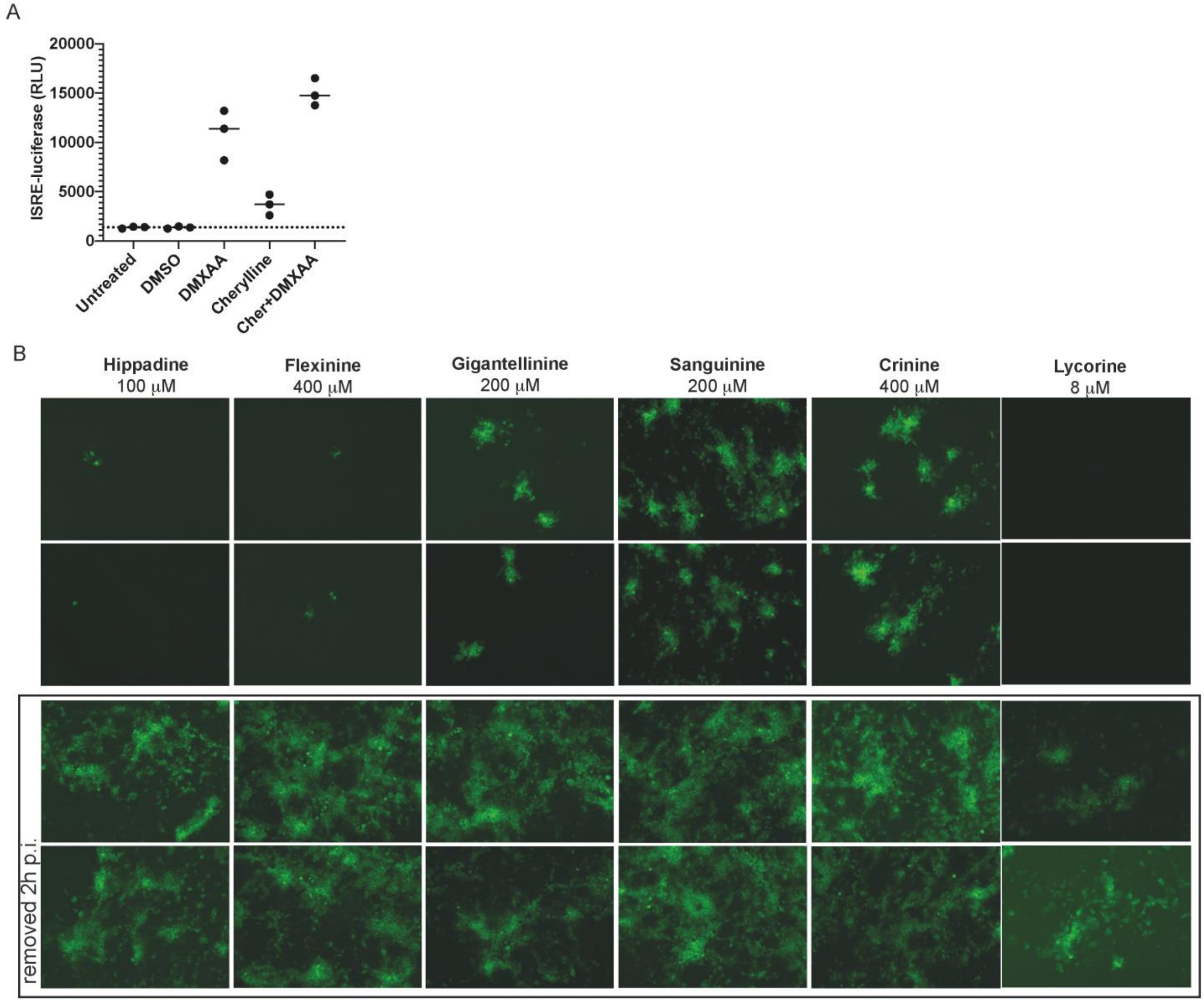

Fig. S3. Screening of AAs activity on virions and on viral entry. A. LL71 cells were treated with 50 μM of cherylline (cher), matched concentrations of DMSO, medium only or with DMXAA (a STING activator) as positive control. Luciferase activity under the promoter of IFN-sensitive response element (ISRE) was measured 24 hours after treatment by luminescence. B. Infectivity of $DENV_{GFP}$ in Huh7 cells continuously treated with AAs compared to cells treated only for 2h p.i.. Two representative pictures of each condition taken with a 5X objective on an inverted microscope system are shown.

Supplementary Figure S4.

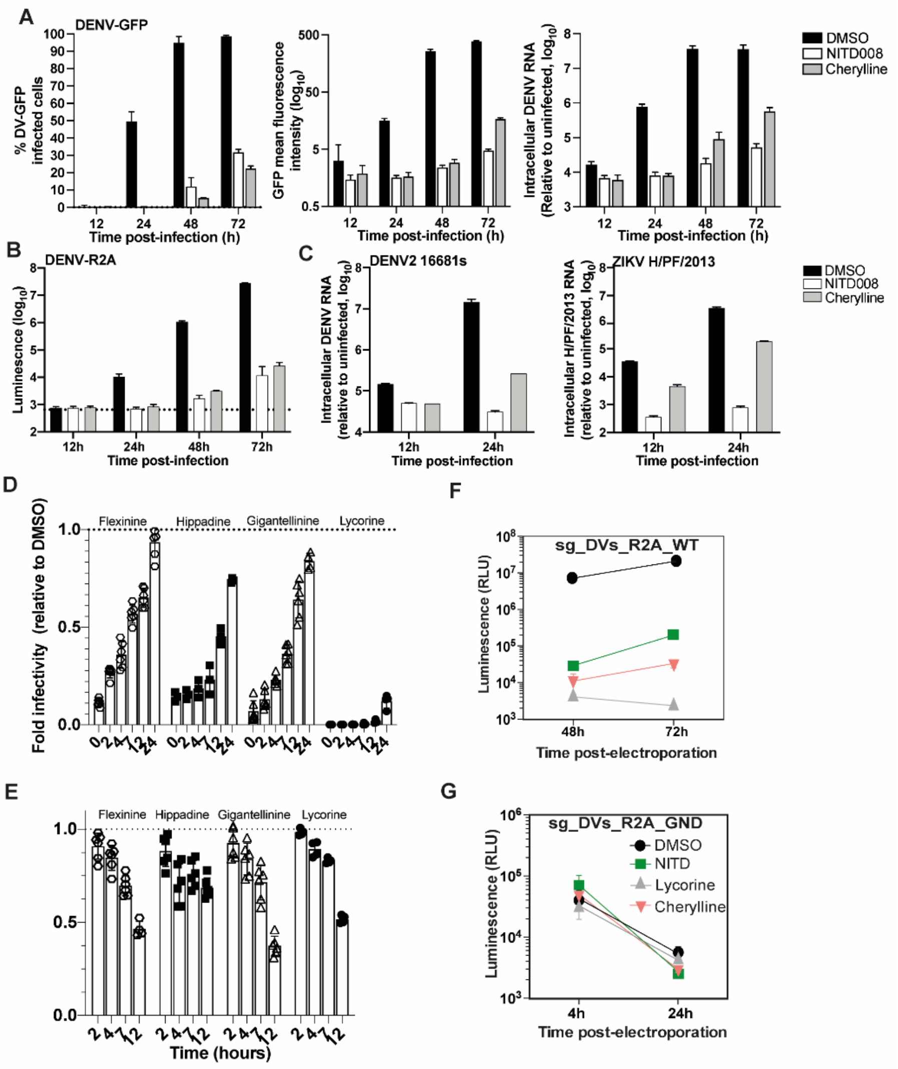

Fig. S4. Viral life cycle steps targeted by AAs. A. Huh7 cells infected with $DENV_{GFP}$ at MOI=0.5 were treated with DMSO as negative control (black), 5 µM NITD008 as positive control (white), or 25 µM of cherylline (gray). Infection levels were assessed as both the % of GFP^+ cells and as the intensity of GFP fluorescence by flow cytometry at 12, 24, 48 and 72 h p.i.. B. Huh7.5 cells were infected with $DENV_{R2A}$ (MOI = 0.005) and treated with 10 µM of NITD008 or 50 µM cherylline. Luminescence was measured at 12, 24, 48 and 72 h p.i. C. Viral RNA levels were monitored by RT-qPCR following RNA extraction at 12 and 24 h.p.i. in $DENV_{GFP}$ (MOI=0.5, Huh7; cherylline = 25 µM, NITD008 = 5 µM), WT DENV 16881s and WT ZIKV H/PF/2013 (MOI=2, Huh7.5, cherylline = 50µM, NITD008 = 10 µM) infected cells. D. Time-of-drug addition of lycorine, hippadine, gigantellinine and flexinine. E. Time-of-drug removal. F. Luciferase levels were quantified at 48 and 72 h post-electroporation (p.e.) with sg-DVs_R2A_WT and normalized over levels detected in DMSO-treated. G. Luciferase levels were quantified at 4 h and 24 h p.e. with RdRp deficient sg-DVs_R2A_GND and normalized over levels in cells treated with DMSO.

Table S1. Ligands from the PDB (Protein Data Bank) database uncovered as similar to cherylline with the SwissSimilarity tool.

		SwissSimilarity (PDB)	
	PDB	Name	Score
hit1	Ren	S-reticuline	0.93
hit2	MS5	7-methoxy-2-(3-methoxybenzyl)-1,2,3,4-tetrahydroisoquinolin-6-yl sulfamate	0.71
hit3	B2Q	(2S,3R,11bR)-3-butyl-9,10-dimethoxy-1,3,4,6,7,11b-hexahydro-2H-pyrido[2,1-a]isoquinolin-2-amine	0.59
hit4	3K8	14aR)-2,3,6-trimethoxy-11,12,13,14,14a,15-hexahydro-9H-dibenzo[f,h]pyrido[1,2-b]isoquinoline	0.41

Table S2. Cherylline's *in silico* predicted targets using shape or pharmacophore screening.

	**Shape screening (SwissTargetPrediction)**			**Shape screening (ChemMapper)**			**Pharmacophore screening (PharmMapper)**		
**Hit**	Uniprot	Name	Score	Uniprot	name	Score	PDB	name	z'-score
1	**Q01959**	dopamine transporter	0.49	**Q00535**	Cell division protein kinase 5	1	**2ZKC**	Estrogen-related receptor gamma	3.87
2	**P31645**	Serotonine transporter	0.34	**P03372**	Estrogen receptor	0.95	**1QUG**	Lyzozyme	1.68
3	**P23975**	Norepinephrine transporter	0.31	**Q04828**	aldo-keto reductase family 1 member C1	0.92	**1OKL**	Carbonic anhydrase 2	1.57
4	**P21728**	Dopamine D1 receptor	0.26	**P21918**	D(1B) dopamine receptor	0	**1JV4**	Major urinary protein 2	1.52

Table S3. Admet properties of Cherylline calculated by the SwissAdme tool.

	Physicochemical properties									Druglikeness	
	**Formul1**	**molecular weight (g/mol)**	**Heavy atoms**	**Aromatic heavy atoms**	**Rotating bounds**	**H bonds acceptors**	**H bond donor**	**Solubility logS**	**lpophilicity logP o/w**	**Lipinski – violation**	**Biodisponibility score**
**Cherylline**	C17H19NO3	285.34	21	12	2	4	2	-3.48	2.70	Y ; 0	0.55
**Gigantelline**	C18H21NO3	299.36	22	12	3	4	1	-3.68	2.89	Y ; 0	0.55
**Gigantellinine**	C18H21NO4	315.36	23	12	3	5	2	-3.55	3.03	Y ; 0	0.55
**Crinine**	C16H17NO3	271.31	20	6	0	4	1	-2.82	2.57	Y ; 0	0.55
**Gigancrinine**	C16H17NO4	287.31	21	6	0	5	1	-2.39	2.57	Y ; 0	0.55
**Flexinine**	C16H17NO4	287.31	21	6	0	5	1	-2.39	2.64	Y ; 0	0.55
**Lycorine**	C16H17NO4	287.31	21	6	0	5	2	-1.82	2.24	Y ; 0	0.55
**Hippadine**	C16H19NO3	263.25	20	16	0	3	0	-3.99	2.58	Y ; 0	0.55
**Sanguinine**	C16H19NO3	273.33	20	6	0	4	2	-2.71	2.12	Y ; 0	0.55

Y : yes ; N : no. Cherylline isomeric smiles code: CN1C[C@H](C2=CC(=C(C=C2C1)O)OC)C3=CC=C(C=C3)O was used as entry.
